# Factors associated with medication adherence among people with diabetes mellitus in poor urban areas of Cambodia: A cross-sectional study

**DOI:** 10.1371/journal.pone.0225000

**Published:** 2019-11-19

**Authors:** Akiyo Nonogaki, Hen Heang, Siyan Yi, Maurits van Pelt, Hiroko Yamashina, Chie Taniguchi, Tomoko Nishida, Hisataka Sakakibara

**Affiliations:** 1 Department of Nursing, Graduate School of Medicine, Nagoya University, Nagoya, Aichi, Japan; 2 MoPoTsyo Patient Information Centre, Phnom Penh, Cambodia; 3 KHANA Center for Population Health Research, Phnom Penh, Cambodia; 4 Saw Swee Hock School of Public Health, National University of Singapore and National University Health System, Singapore, Singapore; 5 Center for Global Health Research, Touro University California, Vallejo, CA, United States of America; 6 Faculty of Health Sciences, Hokkaido University, Sapporo, Hokkaido, Japan; 7 College of Nursing, Aichi Medical University, Nagakute, Aichi, Japan; 8 Department of Nursing, Sugiyama Jogakuen University, Nagoya, Aichi, Japan; 9 School of Nursing, Ichinomiya Kenshin College, Ichinomiya, Aichi, Japan; 10 Nagoya University, Nagoya, Aichi, Japan; Institute of Economic Growth, INDIA

## Abstract

**Background:**

In Cambodia, the age-standardized prevalence of diabetes mellitus has increased in both men and women. The main objective of this study was to identify factors associated with diabetes medication adherence among people with diabetes mellitus in poor urban areas of Phnom Penh, Cambodia.

**Methods:**

A cross-sectional study was conducted in 2017 using a structured questionnaire for face-to-face interviews by trained interviewers. The participants were people with diabetes mellitus who were the active members of a peer educator network, lived in poor urban areas of Phnom Penh, and attended weekly educational sessions during the survey period. Diabetes medication adherence was measured using four items of modified Morisky Medication Adherence Scale. Participants were classified into two groups based on their adherence score: 0 (high adherence) and from 1 to 4 (medium or low adherence). Sociodemographic characteristics; medical history; accessibility to health services; and knowledge, attitude, and practices related to diabetes mellitus were examined. A multiple logistic regression analysis was conducted adjusting for sex, age, marital status, and education levels.

**Results:**

Data from 773 people with diabetes were included in the analyses. Of the total, 49.3% had a high level of diabetes medication adherence. A high level of adherence was associated with higher family income (≥50 USD per month) (adjusted odds ratio [AOR] = 5.00, 95% confidence interval [CI] = 2.25–11.08), absence of diabetes mellitus-related complications (AOR = 1.66, 95% CI = 1.19–2.32), use of health services more than once per month (AOR = 2.87, 95% CI = 1.64–5.04), following special diet for diabetes mellitus (AOR = 1.81, 95% CI = 1.17–2.81), and absence of alcohol consumption (AOR = 13.67, 95% CI = 2.86–65.34).

**Conclusions:**

High diabetes medication adherence was associated with better family economic conditions, absence of diabetes mellitus-related complications, and healthy behaviors. It would be crucial to improve affordable access to regular follow-ups including promotion of healthy behaviors through health education and control of diabetes mellitus-related complications.

## Introduction

In recent years, non-communicable diseases (NCDs) have been the cause of majority of deaths in the developing countries, where the burden of NCDs is greater than that of communicable diseases [1]. NCDs are the primary cause of catastrophic and impoverishing health expenses [2]. A recent literature review has concluded that health system response to NCDs in the Asia-Pacific region is weak [3].

In Cambodia, according to Institute for Health Metrics and Evaluation, the estimated number of deaths caused by diabetes mellitus in 2017 was reported to be 2,756, which was 1.4 times of the number in 1990 [4]. The International Diabetes Federation predicted that the number of adults aged from 20 to 79 years with diabetes mellitus in Cambodia was 246,200 in 2017 [5]. The World Health Organization (WHO) estimated that the number of people with diabetes mellitus in Cambodia would reach approximately 317,000 in 2030, which is about three times of the number in 2000 (*n* = 110,000) [6]. In addition, it is predicted that about half of the people with diabetes mellitus would remain undiagnosed and untreated [7]. Despite considerable improvements in the health sector, access to affordable and effective health care services in the country remains a problem, particularly in the poor and vulnerable populations [8]. About a quarter of all adults in a suburban community had some degree of glucose intolerance, even though the Cambodian society is relatively poor, and their lifestyle is fairly traditional, based on international standards [9]. Therefore, the improvement of multifaceted strategies is crucial in promoting the early identification of diabetes mellitus, the initiation of treatment, and the development of higher-quality treatments.

In resource-limited settings, such as Cambodia, laboratory and drug costs could act as barriers in meeting treatment goals [10]. According to the Cambodia STEPwise approach to chronic disease risk factor surveillance 2010, the proportion of individuals who were previously diagnosed with diabetes mellitus and were taking oral antidiabetic drugs was 58.2% [11]. In Cambodia, diabetes mellitus age-adjusted comparative prevalence was one of the lowest (3.0%) in the Western Pacific region [12]. However, the estimated proportion of individuals whose deaths can be attributable to diabetes mellitus before the age of 60 years was between 60% to 80% [12].

Medication adherence is defined by the WHO as "the extent to which a person’s behavior corresponds with agreed recommendations from a health care provider" [13]. Adherence to long-term therapies is simultaneously influenced by several factors, including social and economic factors, health care providers/system, characteristics of the disease, disease therapies, and patient-related factors [13]. These factors are linked to intentional and unintentional non-adherence. Unintentional non-adherence arises from capacity and resource limitations. The limitations prevent patients from implementing their decisions to follow treatment recommendations and sometimes involves individual constraints [14]. Poor adherence to medication regimens contributes to the substantial worsening of the disease, increased health care costs, and deaths [15]. In contrast, higher medication adherence results in lower health care costs and better diabetes mellitus self-management [16, 17]. Therefore, medication adherence could be used as an indicator for self-management in individuals with diabetes mellitus. The properties of the Morisky’s medication adherence scale were designed to facilitate the identification of problems and barriers to adequate compliance [18]. Previous studies have shown several factors associated with medication adherence including age, job, and self-perception of disease [16, 19, 20]. However, the factors are not well-investigated in least developed countries such as Cambodia, where scientific research is still emerging after decades of civil wars and social economic constraints [21, 22].

The present study aimed (1) to reveal the state of medication adherence among active members of a peer educator network with diabetes mellitus in poor urban areas of Phnom Penh and (2) to identify factors associated with diabetes medication adherence in this population. Findings from this study will contribute to the development of effective strategies and programs that improve health care for diabetes mellitus in Cambodia as well as in other resource-poor settings.

## Methods

### Study design

This was a cross-sectional study that used a structured questionnaire for face-to-face interviews conducted by trained interviewers.

### Ethical consideration

A verbal informed consent was obtained from all participants by the trained interviewers prior to data collection considering the low literacy rate among prospective participants. The right to decline or quit the research at any time without any consequences was explained to the participants. This study was approved by the Ethical Committee of the Graduate School of Medicine, Nagoya University (16–137), and the National Ethics Committee for Health Research, Ministry of Health, Cambodia (114 NECHR).

### Study setting

Patient Information Centre (MoPoTsyo) is a Cambodian non-governmental organization (NGO), which was established in 2004. The organization set-up the community-based “Peer-Educator Network” of individuals with diabetes mellitus in 2005 [23, 24, 25, 26, 27]. The network aims to improve access to reliable information and health care for chronic diseases including diabetes mellitus and hypertension among those who are in socioeconomic constraints. Peer educators and the members can share knowledge and skills with each other via weekly group sessions at the home of a peer educator who also lives in the same community. MoPoTsyo uses the peer educators to facilitate the delivery of affordable medical services for its members. The services are delivered in collaboration with the public health facilities, private pharmacies on contract, and at the home of the peer educators. Four types of services include biochemistry laboratory profiles (blood and urine tests), medical consultation sessions, a revolving drug fund for routine prescription of medications, and follow-up services by the peer educators. The follow-up services are to provide ongoing diabetes education and support, and to check blood glucose levels and blood pressure at the home of peer educators. The members are also encouraged to use urine glucose strips at home to self-monitor their diabetes mellitus control [26]. In general, urine testing is less optimal than blood glucose testing in clinical settings. However, MoPoTsyo provides both blood and urine testing services to promote self-management of diabetes mellitus sustainably. The blood glucose testing is often not affordable for the poor because of the cost and required self-testing skills. On the other hand, urine glucose strip testing is much more affordable and easier to do by the members themselves at home. Therefore, MoPoTsyo uses urine glucose self-testing as a tool for self-screening for diabetes mellitus and self-management of diabetes mellitus. For people who are likely to have diabetes mellitus, blood glucose testing were performed to confirm the diagnosis. MoPoTsyo ensures that the network and its contracted public and private health service providers have sufficient supplies of basic equipment and consumables, including insulin and oral drugs for controlling blood sugar, blood pressure, and lipid levels; urine test strips; scales for measuring body weight; and automated blood pressure devices.

### Participants

People with diabetes mellitus who were members of the Peer-Educator Network and lived in the poor urban areas under the coverage of the network in Phnom Penh were included in the study. The total number of active members in the whole country was 13,232 in 2017. An active member was defined as a person with diabetes mellitus who had used any one of the four services provided by MoPoTsyo within the past 12 months. MoPoTsyo’s peer educators were active in only five poor urban areas (Anlong Kgan, Boeng Kak 2, Srac Chork, Boeng Salang, Borey Santepheap 2) of Phnom Penh. All five poor urban areas were included in the study. The total number of active members in the five poor urban areas at the start of the study was 1,507. Men and women who were aged older than 20 years, Khmer speaking, and taking oral antidiabetic drugs were enrolled in the study regardless of whether they were on injectable insulin treatment. In total, 853 members joined weekly educational sessions during the survey period of two months and consented to participate in the survey.

### Survey procedures

A structured interview was conducted in Khmer. Five local interviewers who were literate and able to communicate in English were chosen by a Monitoring Officer employed by MoPoTsyo. The interviewers received a training, which included pre-testing, by the authors at MoPoTsyo’s office for two days according to an original survey manual. The training contents were used to validate the study outlines and the meaning of all measurements, particularly specific medical terms and human research ethical issues. The participants were compensated for their time and effort with a blood sugar test for free. A daily wage was provided to each interviewer. The survey period lasted for two months from May to July 2017. The peer educators called their active members to join a weekly educational session at their home using a registered member list that was provided by MoPoTsyo. Face-to-face interviews were conducted individually after the session.

### Questionnaire development

The quality assurance in questionnaire development processes included back translation between English and Khmer. The pretesting of the questionnaire was performed in ten members of the Peer-Educator Network who joined a weekly educational session at MoPoTsyo’s headquarter office during the interviewers’ training day. These ten members were later excluded from the main study.

### Measurements

The questionnaire collected information on diabetes medication adherence, sociodemographic characteristics, medical history, accessibility to health services, and state of health management as well as knowledge, attitudes, and practices of people with diabetes mellitus.

Adherence to diabetes medication was measured using four items in Khmer language adapted from the Morisky Medication Adherence Scale (MMAS-4) [18, 28, 29, 30, 31]. This scale is protected by U.S. and International Trademark and Copyright laws. A Retroactive and Corrective, Morisky Widget^TM^ MMAS-4^TM^ License Agreement was made between Nagoya University and MMAS Research LLC. A score of 0 was provided to those who answered no, and 1 to those who answered yes. The scores of the items were summed up to define three levels of adherence: 0 (high adherence), from 1 to 2 (medium adherence), and from 3 to 4 (low adherence) [32]. Then, the adherence levels were reclassified into two groups: 0 (high adherence) and from 1 to 4 (medium or low adherence) [19]. The reason is that the proportion of participants who had low adherence was only 17.2%, and the total proportion of participants who had medium or low adherence accounted for half of the total number of participants (50.7%). Therefore, the medium or low adherence groups were combined as a reference group to identify factors associated with a high level of diabetes medication adherence. The Cronbach’s coefficient alpha of the scale in this study was 0.78. The content validity was assured by multiple researchers working in diabetes mellitus in Cambodia.

Sociodemographic characteristics included sex, age, survey area, marital status (married, single, widow/widower, separated), number of household members (1–2, 3–5, ≥6), employment status (employed, self-employed, household/unemployed), education levels (no formal schooling, some primary, completed primary, completed secondary or higher), literacy level (illiterate, read only, read and write), monthly family income in US dollars (<50, 50–99, 100–249, 250–499, ≥500), possession of a ID poor card, and access to social health protection schemes [2]. Information about diagnosed diseases, diagnosed diabetes mellitus-related complications, and family history of diabetes mellitus were collected to assess the medical history of the participants. To assess accessibility to health facilities, we collected information on the kind of and distance to the nearest health facility, regularly used health facilities, and frequency of using the health facilities [33]. Health facilities were categorized into five types: 1) MoPoTsyo, 2) health center/health post, 3) public hospital, 4) private health facilities, and 5) Kru Khmer (traditional medicine and healers). MoPoTsyo included the headquarter office and five peer educators’ homes. Accessibility to health information included the frequency of joining a health education group session conducted by peer educators during the past one year, availability of health information, and the most trusted source of health information [34]. State of health management included the frequency of measuring body weight, checking of blood pressure and blood sugar level, and conducting urine strip and blood tests. These examinations are made available to the participants by MoPoTsyo via the peer educators in the communities, or at its headquarter office in Phnom Penh.

Measures to evaluate knowledge, attitudes, and practices of the participants were adapted from the “Guide to developing knowledge, attitude, and practice survey” [35]. Knowledge was measured using nine items associated with general knowledge on diabetes mellitus (diet patterns, exercises, alcohol consumption habit, smoking habit, and complications), which were modified based on the diabetes mellitus knowledge test including 17 items developed by MoPoTsyo [36]. Attitude was measured using two items, which included the following: 1) When you have diabetes, should you seek treatment? and 2) Do you think you can influence diabetes by yourself? [36, 37]. Practice was measured using seven yes/no questions associated with the general health practice of individuals with diabetes mellitus (following special diet for diabetes mellitus, regular exercise, smoking habit, alcohol consumption habit, foot care, and counseling conducted by a health worker) [38, 39]. The following special diet for diabetes mellitus was defined following the MoPoTsyo Food Pyramid for Diabetes with Khmer daily food. The A3 poster is provided to every member by the peer educators to put on the wall in their home. Knowledge and attitude items were associated with three categorical response options that included yes, no, or don’t know. For the knowledge, attitude, and practice items, 1 point was provided for every correct or positive answer. The score ranged from 0 to 9 for knowledge, 0 to 2 for attitude, and 0 to 7 for practice. The total knowledge, attitude, and practice score (KAP score) was the sum of these three scores. The mean of knowledge, attitude, practice, and KAP scores were used for the analyses.

### Data analyses

IBM Statistical Package for the Social Sciences (SPSS) software version 24.0 was used in the data analyses. A p-value of 0.05 was used to determine statistical significance level. Descriptive analyses were performed on all variables using the Chi-square test and Fisher’s exact test (when a cell count smaller than 5) to compare characteristics of participants in the high diabetes medication adherence group and the medium or low adherence group. The practice items were analyzed one by one to accurately evaluate health behavior. Student’s *t* test was used to compare the mean score of knowledge, attitudes, practices, and KAP of participants with a high level of diabetes medication adherence and those with a medium or low level of adherence group.

Multiple logistic regression analysis was conducted to identify factors independently associated with levels of diabetes medication adherence. The model was adjusted for sociodemographic characteristics including sex, age, marital status, and education levels, which were found to be significantly associated with diabetes medication adherence in previous studies [40, 41] and all other variables, which were significantly associated with levels of adherence in bivariate analyses. The coefficient of determination between frequency of measuring body weight and frequency of checking blood pressure was 0.382, which indicated that the relation was weak. Each coefficient of determination between frequency of using health facilities and having diabetes mellitus-related complications, frequency of measuring body weight, and checking blood pressure was less than 0.1. Adjusted odds ratios (AORs) with 95% confidence intervals (CI) were used to describe the strength of the association between the independent and dependent variables (level of diabetes medication adherence). Attitude score was not considered as an independent variable because 96.9% of the participants got the maximum score. The practice and KAP scores were not used as an independent variable in the multiple logistic regression analysis because each practice item was analyzed as an independent variable.

## Results

### General characteristics of the study sample

Of the 853 participants, data from 773 participants with complete answers were included in the analyses (completion rate: 90.6%). Eighty participants were excluded from the analyses due to the following reasons: 17 of them participated in the survey more than once, and 63 questionnaires were incomplete. The characteristics of the participants are shown in Table 1. Of the total 773 participants, 57.6% were women, and 80.2% were married. Participants aged less than 45 years old were 9.2%. The proportion of unemployed participants was approximately half (52.3%). Most of the participants (98.3%) were not covered by any social health protection scheme. About one in ten (12.8%) were taking both oral and injecting anti-diabetic drugs. The proportion of participants who had a high level of diabetes medication adherence was 49.3%.

**Table 1:** General characteristics of the study sample (n = 773).

Variables	n	%	Variables	n	%
Sex			Treatment		
Man	328	42.4	Oral anti-diabetic drugs	674	87.2
Woman	445	57.6	Oral anti-diabetic drugs & Insulin	99	12.8
Age			Medication adherence		
≤44	71	9.2	High	381	49.3
45–54	202	26.1	Medium/Low	392	50.7
55–64	297	38.4	Social health protection scheme		
≥65	203	26.3	Have	13	1.7
Marital status			Don’t have	760	98.3
Married	620	80.2	Nearest health facility		
Others	153	19.8	Health center/Health Post	68	8.8
Household numbers			Public hospital	152	19.6
1–2	91	11.8	Private health facilities	260	33.5
3–5	416	53.8	MoPoTsyo	295	38.1
≥6	266	34.4	Regularly use for a health facility		
Employmentstatus			Don’t use	1	0.1
Employed	168	21.7	Health center/Health Post	3	0.4
Self-employed	201	26.0	Public hospital	11	1.4
Household/ Unemployed	404	52.3	Private health facilities	14	1.8
Education levels			MoPoTsyo	744	96.2
No formal schooling	109	14.1	Where to measure body weight		
Some primary	204	26.4	Don't/Unknown	0	0.0
Completed primary	230	29.8	Home	42	5.4
Completed secondary/higher	230	29.8	Health facilities except MoPoTsyo	8	1.0
Literacy			MoPoTsyo	723	93.5
Illiterate	118	15.3	Where to check blood pressure		
Read only	15	1.9	Don't/Unknown	2	0.3
Read and write	640	82.8	Home	57	7.4
Family income(USD/month)			Health facilities except MoPoTsyo	10	1.3
<50	109	14.1	MoPoTsyo	704	91.1
50–99	64	8.3	Where to check blood sugar		
100–249	308	39.8	Don't/Unknown	9	1.2
250–499	209	27.0	Home	59	7.6
≥500	83	10.7	Health facilities except MoPoTsyo	11	1.4
			MoPoTsyo	694	89.8

### Comparisons of characteristics of participants in levels of diabetes mellitus medication adherence

Table 2 shows the differences between people with a high level of diabetes medication adherence and people with a medium or low level of diabetes medication adherence. The proportion of people with a high level of diabetes medication adherence was significantly higher among participants who were female (53.7%, p = 0.004), were not married (61.4%, p = 0.001), had higher monthly family income (≥50 USD, p≤0.001), did not have diabetes mellitus-related complications (55.7%, p≤0.001), had used health facilities, including the home of a peer educator, for more than once per month (54.0%, p≤0.001), had their body weight measured for more than three times per year (50.8%, p = 0.001), had their blood pressure measured for more than three times per year (50.1%, p = 0.031), followed special diet for diabetes mellitus (51.6%, p = 0.003), were non-smokers (52.8%, p≤0.001), and were non-alcohol drinkers (52.9%, p≤0.001) compared to that in their respective reference group.

**Table 2:** Comparisons of characteristics of participants in levels of diabetes medication adherence (n = 773).

Variables	Diabetes medication adherence level				p
	High		Medium/Low		
	n	%	n	%	
Sex					
Man	142	43.3	186	56.7	0.004
Woman	239	53.7	206	46.3	
Age					
≤44	31	43.7	40	56.3	0.241
45–54	92	45.5	110	54.5	
55–64	159	53.5	138	46.5	
≥65	99	48.8	104	51.2	
Marital status					
Married	287	46.3	333	53.7	0.001
Others	94	61.4	59	38.6	
Households number (person)					
1–2	46	50.5	45	49.5	0.469
3–5	212	51.0	204	49.0	
≥6	123	46.2	143	53.8	
Employment status					
Employed	73	43.5	95	56.5	0.128
Self-employed	96	47.8	105	52.2	
Household/Unemployed	212	52.5	192	47.5	
Education levels					
No formal schooling	58	53.2	51	46.8	0.067
Some primary	114	55.9	90	44.1	
Completed primary	107	46.5	123	53.5	
Completed secondary/higher	102	44.3	128	55.7	
Literacy					
Illiterate	64	54.2	54	45.8	0.469
Read only	8	53.8	7	46.7	
Read & Write	309	48.3	331	51.7	
Family income (USD/month)					
<$50	17	15.6	92	84.4	<0.001
$50–99	40	62.5	24	37.5	
$100–249	162	52.6	146	47.4	
$250–499	120	57.4	89	42.6	
≥$500	42	50.6	41	49.4	
ID poor card					
Don’t have	334	49.9	335	50.1	0.369
Have	47	45.2	57	54.8	
Diagnosed diseases					
Yes	203	47.2	227	52.8	0.195
No	178	51.9	165	48.1	
Diabetes mellitus-related complications					
Yes	116	39.1	181	60.9	<0.001
No	265	55.7	211	44.3	
Family history of diabetes mellitus					
Yes	80	44.2	101	55.8	0.118
No	301	50.8	291	49.2	
Distance to the nearest health facility					
<1km	207	52.3	189	47.7	0.311
1–1.9km	47	44.8	58	55.2	
2–4.9km	75	48.7	79	51.3	
≥5km	52	44.1	66	55.9	
Hospitalized					
Didn't	346	50.5	339	49.5	0.058
Did	35	39.8	53	60.2	
Frequency of using health facilities					
≤1/2months	20	19.0	85	81.0	<0.001
≥1/1month	361	54.0	307	46.0	
Frequency of measuring body weight (within 1 year)					
≤2	11	25.0	33	75.0	0.001
≥3	370	50.8	359	49.2	
Frequency of checking blood pressure (within 1 year)					
≤2	11	31.4	24	68.6	0.031
≥3	370	50.1	368	49.9	
Frequency of checking blood sugar (within 1 year)					
≤2	26	50.0	26	50.0	0.915
≥3	355	49.2	366	50.8	
Following special diet for diabetes mellitus					
Yes	335	51.6	314	48.4	0.003
No	46	37.1	78	62.9	
Regular exercise					
Yes	258	50.3	255	49.7	0.433
No	123	47.3	137	52.7	
Current smoker					
Yes	16	19.5	66	80.5	<0.001
No	365	52.8	326	47.2	
Drinking alcohol					
Yes	2	3.5	55	96.5	<0.001
No	379	52.9	337	47.1	
Care when cutting toe nails					
Yes	366	49.8	369	50.2	0.215
No	15	39.5	23	60.5	
Consultation					
Yes	366	49.5	373	50.5	0.537
No	15	44.1	19	55.9	
Health education					
No	96	47.1	108	52.9	0.080
1-5times	64	42.7	86	57.3	
≥6times	221	52.7	198	47.3	

### Mean score of knowledge, attitude, practice, and KAP

Participants who had a high level of diabetes medication adherence had significantly higher mean scores of knowledge (6.97, p = 0.001), attitudes (1.99, p = 0.005), practices (6.26, p≤0.001), and KAP (15.22, p≤0.001) compared to those who had a medium or low level of diabetes medication adherence (Table 3). Levene’s test indicated unequal variances in mean scores of attitudes (F = 33.752, p≤0.001), practices (F = 40.914, p≤0.001), and KAP (F = 3.843, p = 0.05), so degrees of freedom were adjusted from 771 to 598.2 (attitudes), 724.2 (practices), and 767.4 (KAP).

**Table 3:** Mean score of knowledge, attitude, practice, and KAP among people with a high and a medium/low level of diabetes medication adherence (n-773).

Variables	Diabetes medication adherence level		p
	High	Medium/Low	
	Mean	Mean	
Knowledge score	6.97	6.60	0.001
Attitude score	1.99	1.95	0.004
Practice score	6.26	5.69	<0.001
KAP score	15.22	14.24	<0.001

### Factors associated with a high level of diabetes medication adherence

As shown in Table 4, after adjustment for sex, age, marital status, and education levels, a high level of diabetes medication adherence was significantly associated with family income of more than 50 USD per month (AOR = 5.00, 95%CI = 1.19–2.32), absence of diabetes mellitus-related complications (AOR = 1.66, 95% CI = 1.19–2.32), use of health facilities, including the home of a peer educator, more than once per month (AOR = 2.87, 95% CI = 1.64–5.04), following special diet for diabetes mellitus (AOR = 1.81, 95% CI = 1.17–2.81), and absence of alcohol consumption (AOR = 13.67, 95% CI = 2.86–65.34).

**Table 4:** Factors associated with a high level of diabetes medication adherence (n-773).

Variables	AOR	95%CI		p
Family income(USD/month)				
<$50	1.0(reference)	-		-
$50–99	5.00	2.25	-11.08	<0.001
$100–249	4.20	2.22	-7.95	<0.001
$250–499	5.30	2.68	-10.46	<0.001
≥$500	3.87	1.80	-8.33	0.001
Diabetes mellitus-related complications				
Yes	1.0(reference)	-		-
No	1.66	1.19	-2.32	0.003
Frequency of using health facilities				
≤1/2months	1.0(reference)	-		-
≥1/1month	2.87	1.64	-5.04	<0.001
Frequency of measuring body weight (within 1 year)				
≤2	1.0(reference)	-		-
≥3	2.71	0.98	-7.50	0.056
Frequency of checking blood pressure (within 1 year)				
≤2	1.0(reference)	-		-
≥3	0.63	0.21	-1.89	0.407
Following special diet for diabetes mellitus				
No	1.0(reference)	-		-
Yes	1.81	1.17	-2.81	0.008
Smoking				
Yes	1.0(reference)	-		-
No	0.86	0.40	-1.84	0.691
Alcohol consumption				
Yes	1.0(reference)	-		-
No	13.67	2.86	-65.34	0.001
Knowledge score	1.08	0.97	-1.20	0.180

Adjusted for sex, age, marital status, and education levels

## Discussion

This study revealed the levels of diabetes medication adherence among patients with diabetes mellitus in poor urban areas of Phnom Penh and factors associated with the adherence. Approximately half of the participants had a high level of diabetes medication adherence. A high level of adherence was associated with good health behaviors and family economic conditions. The proportion of participants with a high level of diabetes medication adherence of nearly 50% in this study was lower than that reported in a study in Palestine (58.0%), in which the same measurement and cut-off value was used [19]. However, the rate was in accordance with the average level of adherence to long-term therapy for chronic illnesses in developing countries, which were reported at lower than 50% by WHO [13]. Medication adherence gap among countries resulted from the paucity of health resources and inequities in access to health care [13, 42]. Moreover, medication adherence is multifactorial, and the conclusions about the associated factors differed in the literature [43, 19].

The present study showed that high monthly family income was associated with a high level of diabetes medication adherence. In the lower income group, the cost of medications was associated with the lack of adherence [43, 44]. Participants whose monthly family income was less than 50 USD may not be able to afford the cost of oral antidiabetic drugs due to life-competing financial demands, although MoPoTsyo provides the oral anti-diabetic drugs at lower prices compared to other health providers and pharmacies [45]. Cost-related medication underuse, which is to use less medications than prescribed because of the cost, was observed in people with diabetes who did not have any social health protection schemes or had more comorbidities [46]. In this study, 98.3% of the participants did not have access to any social health protection scheme. However, participants with a higher monthly family income (≥50USD/month) could afford medications by MoPoTsyo, which plays a role in substituting social health protection schemes from the view point of economic burden reduction. Transportation would also be influenced by the same economic reason. High-income families could afford transportation. Thus, the distance to health facilities might not directly affect diabetes medication adherence for the high-income families. In addition, the participants were members who could join the regular educational session by MoPoTsyo. This proactive attitude and accessibility to the services offered by MoPoTsyo might weaken sociodemographic and geographic influences on medication adherence. These characteristics may be the reasons why this study did not show statistically significant differences between the sociodemographic factors, except for monthly income, and adherence. An earlier study has indicated that the associated factors differed and had various forms depending on the treatment component and research population [47].

Use of health facilities, including the home of a peer educator, more than once per month was associated with a high level of diabetes medication adherence. This result is in accordance with those of previous studies showing that regular clinic follow-ups have positive effects on medication adherence [48, 49]. MoPoTsyo collaborates with a hospital to provide continuous services between community settings and health facilities. Then, MoPoTsyo can take a role in regular follow-ups with a reasonable and practical approach to deliver effective diabetes care in a rural area [25]. Regular health facility utilization during follow-ups without economic burden is crucial in increasing medication adherence.

Two healthy practices in daily life including restriction on alcohol consumption and following special diet for diabetes mellitus were associated with a high level of diabetes medication adherence although the statistical stability of the variable of alcohol consumption was weak because of the small sample size. Alcohol consumption has been shown to affect the dampening of impulse control, and individuals who use alcohol are less likely to practice the recommended self-care behaviors, such as regular clinical follow-ups and medication adherence [50]. Moreover, even periodic mild intoxication may cause patients who intend to take prescribed medications to forget to take or refill them [51]. With regard to following special diet for diabetes mellitus, individuals with healthy diet behaviors are more likely to be able to control their blood sugar level [52]. By contrast, poor individuals were reported to lack access to facilities that provide information about dietary habits and prescribed medications [53]. It implies that the improvement in medication adherence with the promotion of restriction on alcohol consumption and healthy diet might result in sustainable self-management at an affordable cost among people with diabetes mellitus.

Participants who did not have any diabetes mellitus-related complications had a high level of diabetes medication adherence. Complex treatments, such as the intake of multiple drugs with different pharmacological classes for comorbidities, reduced medication adherence [54]. In contrast, poor medication adherence may result in co-morbidities, which are usually observed in individuals with microvascular complications due to diabetes mellitus [55]. Participants with absence of diabetes mellitus-related complications should be required to take less medication. Thus, their diabetes medication adherence might increase, and the risk of developing comorbidities may decrease.

### Limitations

This study has several limitations. First, as members of a Peer-Educator Network, all study participants might have higher diabetes medication adherence and better knowledge, behavior, and management practices for diabetes mellitus than non-members because they were supported by a community-based organization, and they could join weekly educational sessions by themselves. The members who did not participate in this study might be less likely to join the weekly educational sessions or less interest in medication adherence and self-management. Thus, the study results may not be representative of typical poor individuals with diabetes mellitus. Second, the study results, particularly family income and items associated with healthy behaviors, could be susceptible to social desirability bias because the data were collected through face-to-face interviews. Third, the number of participants with diabetes mellitus-related complications and those with family members who were diagnosed with diabetes mellitus may be underestimated because the data were not collected from medical records and some participants and their family members might be undiagnosed owing to poor accessibility to medical services. Fourth, the validity of MMAS-4 in Khmer language was not known and needs to be assessed by future researches. Finally, the causal relationship between the identified factors with diabetes medication adherence could not be concluded because of the cross-sectional design of the study.

Future studies that follow-up the participants who had different levels of diabetes medication adherence and assess the patients’ health conditions clinically in parallel with diabetes medication adherence are recommended. In addition, it is expected to reveal the influence of underlying traits resulting from individual economic status, rather than family income, which might be the economic barriers to access medication and healthcare services and drive the associated factors with high diabetes medication adherence.

## Conclusions

Half of the participants had a high level of diabetes medication adherence. The identified factors associated with diabetes medication adherence among participants in this study included a higher monthly family income of than 50 USD, regular follow-ups at health facilities including the community-based peer educator group, absence of alcohol consumption, following special diet for diabetes mellitus, and absence of diabetes mellitus-related complications. It would be effective to improve affordable access to regular follow-ups including promotion of heathy behaviors and regular use of health and education services. This study could infer that people who have the lowest income are at increased risk of worsening symptoms due to lack of adherence and detrimental lifestyle behaviors and need to be assisted. In the future, the national health care system should be capable of generating and sustaining capacities of diabetes educators like MoPoTsyo’s peer educators. In addition, the coverage by the social health protection scheme should be expanded to improve the accessibility to medical and healthcare services following the innovative models developed by community-based organizations such as MoPoTsyo.

## Supporting information

S1 Checklist STROBE_checklist_cross-sectional_AkiyoNonogaki. (DOC)

S1 Appendix Questionnaire(English). (DOCX)

S2 Appendix Questionnaire(Khmer). (DOCX)

S1 Dataset minimal data. (XLSX)
