# Supplementary material for: Factors associated with medication adherence among people with diabetes mellitus in poor urban areas of Cambodia: A cross-sectional study
Source: PLoS One. 2019 Nov 19;14(11):e0225000. doi: 10.1371/journal.pone.0225000 (PMC6863566; doi:10.1371/journal.pone.0225000)
Supplement: S1 Checklist — (DOC) [file pone.0225000.s001.doc]

STROBE Statement—Checklist of items that should be included in reports of ***cross-sectional studies***

|  | Item No | Available section |
| --- | --- | --- |
| **Title and abstract** | 1 | (*a*) The study design ‘a cross-sectional study’ was mentioned in the title and Methods in the Abstract, first sentence. |
|  |  | (*b*) Provided in the abstract |
| Introduction | | |
| Background/rationale | 2 | Whole background |
| Objectives | 3 | Background paragraph 5 |
| Methods | | |
| Study design | 4 | ‘Study design’ in the Methods |
| Setting | 5 | The setting in the ‘Study setting’ in the Methods  The location in the ‘Participants’ in the Methods  Relevant dates, including periods of recruitment in the 9^th^ sentence in the ‘Participants’ in the Methods  Data collection in the 9^th^ sentence in the‘Survey procedures’ in the Methods |
| Participants | 6 | ‘Participants’ in the Methods |
| Variables | 7 | ‘Measurements’ in the Methods |
| Data sources/ measurement | 8* | ‘Measurements’ in the Methods |
| Bias | 9 | Limitations |
| Study size | 10 | ‘Participants’ in the Methods |
| Quantitative variables | 11 | Measurements paragraph 2 in the Methods |
| Statistical methods | 12 | (*a*) Data analyses in the Methods |
|  |  | (*b*) N/A |
|  |  | (*c*) At the end of the ‘Participants’ in the Methods |
|  |  | (*d*) N/A |
|  |  | (*e*) N/A |
| Results | | |
| Participants | 13* | (a) At the end of the ‘Participants’ in the Methods |
|  |  | (b) At the end of the ‘Participants’ in the Methods |
|  |  | (c) N/A |
| Descriptive data | 14* | (a) General characteristics of the study sample in the ‘Results’ and Table 1 |
|  |  | (b) At the end of the ‘Participants’ in the Methods |
| Outcome data | 15* | General characteristics of the study sample in the ‘Results’ and Table 1 |
| Main results | 16 | (*a*)  Data analyses paragraph 2 in the Methods  Table 4  ‘Factors associated with high medication adherence’ in the Results |
|  |  | (*b*) N/A |
|  |  | (*c*) N/A |
| Other analyses | 17 | N/A |
| Discussion | | |
| Key results | 18 | Discussion paragraph 1 |
| Limitations | 19 | Limitations |
| Interpretation | 20 | Conclusions |
| Generalisability | 21 | Limitations |
| Other information | | |
| Funding | 22 | Funding |

*Give information separately for exposed and unexposed groups.

**Note:** An Explanation and Elaboration article discusses each checklist item and gives methodological background and published examples of transparent reporting. The STROBE checklist is best used in conjunction with this article (freely available on the Web sites of PLoS Medicine at http://www.plosmedicine.org/, Annals of Internal Medicine at http://www.annals.org/, and Epidemiology at http://www.epidem.com/). Information on the STROBE Initiative is available at www.strobe-statement.org.
