# Supplementary material for: Factors associated with medication adherence among people with diabetes mellitus in poor urban areas of Cambodia: A cross-sectional study
Source: PLoS One. 2019 Nov 19;14(11):e0225000. doi: 10.1371/journal.pone.0225000 (PMC6863566; doi:10.1371/journal.pone.0225000)
Supplement: S1 Appendix — (DOCX) [file pone.0225000.s002.docx]

1. Personal profiles

1. Sex

①Male　②Female

1. Age

①Less than 25　②25-34　③35-44　④45-54　⑤55-64　⑥65-74　⑦75-84　⑧More than 85

1. Living area
2. Anlong Kgan　②Boeng Kak 2　③Srac Chork　④Boeng Salang　⑤Borey Santepheap 2
3. What is your nationality?

①Khmer　②Others

1. What is your marital status?

①Married　②Single　③Widower/Widow　④Separate

1. How many members are in your households?

①1-2members　②3-5members　③6-8members　④9members<

1. Who lives with you at the moment?（Multiples responses allowed）

①Father　②Mother　③Spouse　④Son/Daughter　⑤Brother/Sister　⑥Relative　⑦Other（　 　　　　）

1. What is your current employment status?

①Employed　②Self-employed business　③Household tasks/Unemployed　/Retired/Monk　④Others（　　　　　　　）

1. What level of education did you achieve?

①No formal schooling　②Some primary　③Completed primary　④Completed secondary or beyond

1. Can you read and write in Khmer?

①Illiterate　②Read only ③Read and write

1. Do you have ID poor card?

①No　②Yes

1. How much is your monthly family income?

①No income　②<US$50　③US$50-$99　④US$100-$249　⑤US$250-499　⑥More than US$500

1. Have you ever been diagnosed these diseases? （Multiples responses allowed）

①No　②Hypertension　③Cardiovascular diseases　④Tuberculosis　⑤Stroke　⑥Pulmonary diseases　⑦Others( )

1. Have you ever been diagnosed any diabetes-related complications?（Multiples responses allowed）

①No　②Retinopathy　③Neuropathy　④Nephropathy

1. Have your any family members had diabetes?

①No　②Yes

1. What type of social health protection scheme do you have currently besides Peer Educator Network?

①Community-Based Health Insurance　②Health Equity Funds

③Chronic Disease Clinic　④National Social Security Fund(/for Civil servants)　⑤No scheme

2. Access to Health facilities

1. What is the nearest health facility from your house?

①Health center/Health post　②Public hospital　③Private health facilities　④Patient Information Center　⑤Kru Khmer

1. How far is the nearest health facility from your house?

①Less than 1km　②1-1.9km　③2-4.9km　④More than 5km　⑤Not sure

1. Which health facilities do you go regularly at the moment?

①No　②Health center/Health post ③Public hospital　④Private health facilities　⑤Patient Information Center(Including PE’s home) ⑥Kru Khmer

1. How often do you go to a health facility?

①More than once per month　②Once per three months(2-3months)　③Once per six months(4-6months)　④Once per one year(7-12months)　⑤Less than once per one year/Do not go/Only emergency

1. Who accompanied with you when you go to a health facility?

①Only me　②Parent　③Spouse　④Brother/Sister　⑤Son/Daughter　⑥Friend

3. State of medication control for diabetes

1. Do you have any treatment for diabetes?

①None/Unknown　②Oral antidiabetic drugs(OAD)　③Insulin　④OAD & Insulin

**＜Skip to Section 4. if you don’t have any OAD.>**

1. Do you ever forget to take your medicine?

① Yes　② No

1. Are you careless at times about taking your medicine?

① Yes　② No

1. Sometimes if you feel worse when you take the medicine, do you stop taking it?

① Yes　② No

1. When you feel better do you sometimes stop taking your medicine?

① Yes　② No

1. Where did you get the OAD?

①Health center/Health post　②Drug vendor　③Public hospital　④Private health facilities　⑤Patient Information Center　⑥Kru Khmer

4. State of health management

1. Have you taken a urine strip within three months?

①Don’t /Unknown　②Once　③Twice ④More than three times

1. Where/by whom did you take the latest urine strip?

①Don’t/Unknown　②By myself at my home　③Home visiting by a Peer Educator　④Health center/Health post　⑤Public hospital　⑥Private health facilities　⑦Patient Information Center　⑧Others（　　　　　　　　　）

1. Have you measured your body weight within one year? (Excluding interview day)

①Don’t/Unknown　②Once　③Twice　④More than three times

1. Where/by whom did you measure your latest body weight? (Excluding interview day)

①Don’t/Unknown　②By myself at my home　③Home visiting by a Peer Educator　④Health center/Health post　⑤Public hospital　⑥Private health facilities　⑦Patient Information Center　⑧Others（　　　　　　　　　）

1. Have you checked your blood pressure within one year? (Excluding interview day)

①Don’t/Unknown　②Once　③Twice　④More than three times

1. Where/by whom did you check your latest blood pressure? (Excluding interview day)

①Don’t/Unknown　②By myself at my home　③Home visiting by a Peer Educator　④Health center/Health post　⑤Public hospital　⑥Private health facilities　⑦Patient Information Center　⑧Others（　　　　　　　　　）

1. Have you checked your blood sugar level within one year?

①Don’t/Unknown　②Once　③Twice　④More than three times

1. Where/by whom did you check your latest blood sugar level?

①Don’t/Unknown　②By myself at my home　③Home visiting by a Peer Educator　④Health center/Health post　⑤Public hospital　⑥Private health facilities　⑦Patient Information Center　⑧Others（　　　　　　　　　）

1. Have you taken a blood test within one year?

①Don’t/Unknown　②Once　③Twice　④More than three times

1. Where did you take the latest blood test?

①Don’t/Unknown　②Health center/Health post　③Public hospital　④Private health facilities　⑤Patient Information center　⑥Others（　　　　　　　　　）

5. Cost of accessing medical services

1. Have you been hospitalized within one year?

①Don’t/Unknown　② Once　③ Twice ④More than three times

1. How many days were you hospitalized recently? (within one year)

①Don’t/Unknown　②1-3days　③4-6days　④More than 7 days

1. How much did you pay to travel up and down to the health facilities within one month?

①Don’t/Unknown　②<US$１　③US$1-1.9　④US$2-2.9　⑤US$3-3.9　⑥US$4-4.9　⑦US$5<

1. How much did you pay for the routine medical services within one month?

(Including consultation, examinations, medication)

①Don’t/Unknown　②<US$１　③US$1-2.9　④US$3-4.9　⑤US$5-6.9　⑥US$7-9.9　⑦US$10<

6. Knowledge of diabetes

1. I can eat many eggs as I want.

①True　②False　③Don’t know

1. I can eat many fish as I want.

①True　②False　③Don’t know

1. I can eat beans as I want. (Excluding sweet dessert)

①True　②False　③Don’t know

1. I can eat as much rice as I want.

①True　②False　③Don’t know

1. I can eat as much bread as I want.

①True　②False　③Don’t know

1. Exercising regularly can help control diabetes.

①True　②False　③Don’t know

1. Smoking any tobacco product such as cigarettes, cigars, or pipes influence on control diabetes.

①True　②False　③Don’t know

1. Drinking alcohol influence on control diabetes.

①True　②False　③Don’t know

1. Numbness and tingling may be symptoms of nerve disease.

①True　②False　③Don’t know

7. Attitudes related to diabetes

1. When you have diabetes, should you seek treatment?

①Yes　②No　③Don’t know

1. Do you think you can influence diabetes by yourself?

①Yes　②No　③Don’t know

8. Practices related to diabetes

1. Do you follow some special DM diet at the moment?

①Yes　②No

1. Do you exercise regularly at the moment?

①Yes　②No

1. Do you currently smoke any tobacco product?

①Yes　②No

1. Are you a former tobacco product smoker?

①Yes　②No

1. Do you drink alcohol in daily life?

①Yes　②No

1. Have you taken care when cutting toe nails?

①Yes　②No

1. Have you been counseled about diabetes by a health worker within the past three months? (Excluding interview day)

(A health worker includes a Peer Educator, a health volunteer, a doctor, a nurse, and a dietitian.)

①Yes　②No

9. Opportunities of accessing health information

1. Have you joined health education group session by Peer Educators within one year?

①Don’t/Unknown　②Once　③Twice　③Three times　④four times　⑤five times　⑥More than six times

1. How have you gotten health information mainly within one year?

①Family　②Friend　③Peer Educator　④Health volunteer　⑤Health center　⑥Public hospital　⑦Private health facilities　⑧Radio　⑨Television　⑩Posters/Newspapers/Leaflets　⑪Others（　　　　　　　　　）

1. What is the most trusted source of health information?

①Family　②Friend　③Peer Educator　④Health volunteer　⑤Health center　⑥Public hospital　⑦Private health facilities　⑧Radio　⑨Television　⑩Posters/Newspapers/Leaflets　⑪Others（　　　　　　　　　）

This interview is completed. Thank you very much.

10. Copied existing data

1. Registration date in Peer Educator Network

（　　　　　　　　）month（　　　 　）year

1. The urine strip data when the target registered in Peer Educator Network

①－　②±　③＋　④＋＋　⑤＋＋＋　⑥＋＋＋＋

1. The latest urine strip date

（　　　　　　　　）month（　　　 　）year

1. The latest urine strip data

①－　②±　③＋　④＋＋　⑤＋＋＋　⑥＋＋＋＋

1. The latest weight measured date

（　　　　　　　　）month（　　　 　）year

1. The latest Height, & Weight

( )cm, ( )kg

1. The latest blood exam date

（　　　　　　　　）month（　　　 　）year

1. The blood exam data
