# Supplementary material for: Factors associated with medication adherence among people with diabetes mellitus in poor urban areas of Cambodia: A cross-sectional study
Source: PLoS One. 2019 Nov 19;14(11):e0225000. doi: 10.1371/journal.pone.0225000 (PMC6863566; doi:10.1371/journal.pone.0225000)
Supplement: S2 Appendix — (DOCX) [file pone.0225000.s003.docx]

កម្រងសំនួរ​

**អង្កេតស្រាវជ្រាវ ក្រោមប្រធានបទ៖**

“កត្តាពាក់ព័ន្ធនានា អំពីប្រសិទ្ធភាពនៃការគ្រប់គ្រងជំងឺដោយខ្លួនឯង ក្នុងចំនោមអ្នកជំងឺទឹកនោមផ្អែមប្រភេទ២ នៅតាមសហគមន៍ក្រីក្រនានា ក្នុងប្រទេសកម្ពុជា”

**1. អំពីព័ត៌មានផ្ទាល់ខ្លួន**

1. អំពីភេទ៖

① ប្រុស　②​ ស្រី

1. អំពីអាយុ៖

① ក្រោម ២៥ឆ្នាំ　 ② ២៥ - ៣៤ឆ្នាំ ③៣៥-៤៤ឆ្នាំ ④៤៥-៥៤ឆ្នាំ ⑤​៥៥-៦៤ឆ្នាំ　⑥៦៥ - ៧៤ឆ្នាំ ⑦៧៥ - ៨៤ឆ្នាំ ⑧៨៥ឆ្នាំឡើង

1. ទីកន្លែងរស់នៅ៖

① អន្លង់ក្ងាន　②បឹងកក់២　③ស្រះចក　④បឹងសាឡាង 　⑤បូរីសន្តិភាព២

1. តើអ្នកមានសញ្ជាតិអ្វី?

①ខ្មែរ　②ផ្សេងៗ

1. អំពីស្ថានភាពគ្រួសារ៖ What is your marital status?

①មានគ្រួសារ(រៀបការ)　②នៅលីវ　③ផ្សេងៗ

1. តើក្នុងគ្រួសាររបស់អ្នកមានសមាជិកប៉ុន្មាននាក់?

① ១ ទៅ ២ នាក់ ②៣ទៅ ៥​នាក់ ③៦ ទៅ ៨ នាក់　⑦ ៩នាក់ឡើងទៅ

1. តើអ្នកណាខ្លះកំពុងរស់នៅជាមួយអ្នក?（Multiples responses）

①ឳពុក　 ②ម្តាយ　 ③ប្តីប្រពន្ធ　 ④កូនៗ

⑤បងប្អូនបង្កើត　 ⑥សាច់ញាត្តិ　 ⑦ផ្សេងៗ（　.........................）

1. តើបច្ចុប្បន្ន អ្នកកំពុងប្រកបរបរអ្វី?

①ធ្វើការអោយគេ　 ②ធ្វើជំនួញដោយខ្លួនឯង ③ធ្វើការងារផ្ទះ/គ្មានការងារ/និវត្តន៍ជន/ព្រសង្ឃ　④ផ្សេងៗ（...............................）

1. តើអ្នកបានបញ្ចប់ការសិក្សាត្រឹមថ្នាក់ណា?

①មិនបានចូលរៀន ②ថ្នាក់បឋមសិក្សា ③ចប់ថ្នាក់បឋមសិក្សា

④ចប់មធ្យមសិក្សា ឬខ្ពស់ជាងនេះ

1. តើអ្នកអាចអាន ឬសរសេរអក្សរខ្មែរបានដែរ​ ឬទេ?

①អត់ចេះសោះ　 ②ចេះអានបាន ③ចេះអាន​ និងសរសេរបាន

1. តើអ្នក មានប័ណ្ណក្រីក្រដែរ ឬទេ?

①ទេ　 ②បាទ/ចាស៎

1. តើចំណូលប្រចាំខែក្នុងគ្រួសាររបស់អ្នក មានប៉ុន្មាន (ដុល្លា)?

①គ្មានសោះ　 ②ត្រឹមUS$29　 ③ចន្លោះពីUS$30-$69　 ④ចន្លោះពីUS$70-$99　⑤ចាប់ពីUS$100​ឡើង

1. តើអ្នកធ្លាប់ត្រូវបានរកឃើញថា មានជំងឺខាងក្រោមនេះ ដែរ​ ឬទេ នាពេលកន្លងមក?

①គ្មានជំងឺទេ　 ②លើសសំពាធឈាម　 ③លើសជាតិខ្លាញ់　 ④របេង

⑤ដាច់សរសៃឈាម　⑥ជំងឺសួត　 ⑦ផ្សេងៗ(..............................)

1. តើអ្នកធ្លាប់ត្រូវបានរកឃើញថា មានជំងឺឱកាសនិយមខាងក្រោមនេះដែរ​ ឬទេ?（Multiples responses allowed）

①គ្មាន　 ②ជំងឺបាតភ្នែក　③ជំងឺពាក់ព័ន្ធនឹងប្រព័ន្ធប្រសាទ　④ជំងឺតម្រងនោម

1. តើក្នុងគ្រួសារអ្នក មានអ្នកណាកើតជំងឺទឹកនោមផ្អែមដែរ ឬទេ?

①ទេ　 ②បាទ/ចាស៎

1. តើអ្នកបានទទួលផល ឬប្រើសេវារ៉ាប់រងសុខភាពណាមួយ ដែរ ឬទេ?

①ធានារ៉ាប់រងសុខភាពតាមសហគមន៍ ②មូលនិធិសមធម៌ ③សេវារបស់ក្លីនិចឯកទេស

④បណ្តាញមិត្តអប់រំមិត្ត បេឡាជាតិរបបសន្តិសុខសង្គម(មន្ត្រីរាជការ) ⑥គ្មាន

**2. ការទទួលបានសេវាថែទាំព្យាបាលសុខភាព**

1. តើមួយណា ជាកន្លែងផ្តល់សេវាសុខភាពនៅជិតអ្នកជាងគេ?

①ប៉ុស្តិ៍ ឬមណ្ឌលសុខភាព ②មន្ទីរពេទ្យរដ្ឋ　 ③សេវាពេទ្យឯកជន

④មណ្ឌលព័ត៌មានជំងឺ ⑤គ្រូខ្មែរ

1. តើ កន្លែងផ្តល់សេវាសុខភាពនៅជិតអ្នកជាងគេ មានចំងាយប៉ុន្មានគីឡូម៉ែត្រ?

①ក្រោម1km　 ②1-2km 　 ③2-5km　 ④លើសពី​5km 　⑤អ្នកដឹងច្បាស់ទេ

1. តើ កន្លែងផ្តល់សេវាសុខភាពមួយណា ដែលអ្នកទៅរកញឹកញ៉ាប់ជាងគេនាពេលសព្វថ្ងៃនេះ?

①គ្មាន　 ②ប៉ុស្តិ៍ ឬមណ្ឌលសុខភាព ③មន្ទីរពេទ្យរដ្ឋ

④សេវាពេទ្យឯកជន ⑤មណ្ឌលព័ត៌មានជំងឺ　 ⑥គ្រូខ្មែរ

1. តើអ្នកទៅកាន់កន្លែងផ្តល់សេវាសុខភាព ញឹកញ៉ាប់ប៉ុណ្ណាដែរ?

①ច្រើនជាង១ដង/ខែ　 ②៣ខែម្តង ③៦ខែម្តង

④១ឆ្នាំម្តង　 ⑤តិចជាង១ឆ្នាំម្តង/អត់ដែលទៅ/ទៅតែពេលឈឺបន្ទាន់

1. តើអ្នកទៅមើលថែអ្នក(ទៅទ្រាំ) នៅពេលអ្នកទៅពេទ្យ?

①ទៅពេទ្យម្នាក់ឯង ②មា្តយ ឬឳពុក　③ប្តីប្រពន្ធ　④បងប្អូនបង្កើត　⑤កូនៗ　⑥មិត្តភក្តិ

**3. ស្ថានភាពនៃការគ្រប់គ្រងតាមបែបវេជ្ជសាស្រ្តសម្រាប់ជំងឺទឹកនោមផ្អែម**

1. តើអ្នកធ្វើការព្យាបាលជំងឺទឹកនោមផ្អែមរបស់អ្នកដែរ ឬទេ?

①ទេ/អត់ដឹង　②ប្រើថ្នាំលេប(OAD)　③ប្រើអាំងស៊ុយលីន　④ប្រើថ្នាំលេបផង និងអាំងស៊ុយលីនផង

***＜រម្លងទៅសំនួរទី៤ ប្រសិនបើ អ្នកជំងឺមិនបានប្រើថ្នាំលេប>***

1. តើអ្នកធ្លាប់ភ្លេចលេបថ្នាំទឹកនោមផ្អែមរបស់អ្នកដែរ ឬទេ?

① បាទ/ចាស៎ ② ទេ

1. តើអ្នកមានភាពប្រហែស ឬ មិនខ្វល់អំពីពេលត្រូវលេបថ្នាំដែរ ឬទេ?

① បាទ/ចាស៎ ② ទេ

1. ពេលខ្លះ ប្រសិនបើពេលលេបថ្នាំទឹកនោមផ្អែមរបស់អ្នក វាធ្វើអោយអ្នកមិនស្រួលខ្លួន តើអ្នកបានឈប់លេបវាដែរ ឬទេ?

① បាទ/ចាស៎ ② ទេ

1. នៅពេលដែលអ្នក មានអារម្មណ៍ថាធូរស្បើយពីជំងឺទឹកនោមផ្អែមរបស់អ្នកហើយ តើអ្នកបានឈប់លេបថ្នាំដែរ ឬទេ?

① បាទ/ចាស៎ ② ទេ

1. តើអ្នកបានទៅទិញថ្នាំលេប សម្រាប់ទឹកនោមផ្អែមរបស់អ្នក នៅឯណា?

①ប៉ុស្តិ៍ ឬមណ្ឌលសុខភាព ②ឱសថស្ថាន　③តាមមន្ទីរពេទ្យរដ្ឋ　④តាមក្លីនិចឯកជន

⑤មណ្ឌលព័ត៌មានជំងឺ　 ⑥គ្រូខ្មែរ

**4. ស្ថានភាពនៃការគ្រប់គ្រងសុខភាព**

1. តើអ្នកបានប្រើស្រ្តីបតេស្តមើលជាតិស្ករក្នុងទឹកនោម ក្នុងរយៈពេល ៣ខែចុងក្រោយនេះ?

①ទេ/អត់ដឹង　 ②ម្តង　 ③២ដង ④៣ដងឡើង

1. តើអ្នកធ្វើតេស្តជាតិស្ករក្នុងទឹកនោមលើកចុងក្រោយនៅឯណា? ជាមួយអ្នកណា?

①ទេ/អត់ដឹង　 ②ធ្វើខ្លួនឯងនៅផ្ទះ　 ③មិត្តអប់រំមិត្តធ្វើអោយនៅផ្ទះខ្លួនឯង ​④ប៉ុស្តិ៍ ឬមណ្ឌលសុខភាព

⑤មន្ទីរពេទ្យរដ្ឋ ⑥នៅពេទ្យឯកជន　⑦មណ្ឌលព័ត៌មានជំងឺ　 ⑧ផ្សេងៗ（　..........................　）

1. តើអ្នកធ្លាប់បានថ្លឹងគីឡូរបស់អ្នក ក្នុងរយៈពេលមួយឆ្នាំចុងក្រោយនេះ ដែរ​ ឬទេ?

①ទេ/អត់ដឹង　②ម្តង　③២ដង ④៣ដងឡើង

1. តើអ្នកបានថ្លឹងគីឡូរបស់អ្នក លើកចុងក្រោយនៅឯណា? ជាមួយអ្នកណា?

①ទេ/អត់ដឹង　②ធ្វើខ្លួនឯងនៅផ្ទះ　③មិត្តអប់រំមិត្តធ្វើអោយនៅផ្ទះខ្លួនឯង ④ប៉ុស្តិ៍ ឬមណ្ឌលសុខភាព

⑤មន្ទីរពេទ្យរដ្ឋ ⑥នៅពេទ្យឯកជន　⑦មណ្ឌលព័ត៌មានជំងឺ　 ⑧ផ្សេងៗ（　...........................　）

1. តើអ្នកធ្លាប់បានវាស់សំពាធឈាមរបស់អ្នក ក្នុងរយៈពេលមួយឆ្នាំចុងក្រោយនេះ ដែរ​ ឬទេ?

①ទេ/អត់ដឹង　 ②ម្តង　 ③២ដង ④៣ដងឡើង

1. តើអ្នកបានវាស់សំពាធឈាមរបស់អ្នក លើកចុងក្រោយនៅឯណា? ជាមួយអ្នកណា?

①ទេ/អត់ដឹង　②ធ្វើខ្លួនឯងនៅផ្ទះ　③មិត្តអប់រំមិត្តធ្វើអោយនៅផ្ទះខ្លួនឯង ④ប៉ុស្តិ៍ ឬមណ្ឌលសុខភាព

⑤មន្ទីរពេទ្យរដ្ឋ ⑥នៅពេទ្យឯកជន　⑦មណ្ឌលព័ត៌មានជំងឺ　⑧ផ្សេងៗ（　...........................　　）

1. តើអ្នកធ្លាប់បានវាស់ជាតិស្ករក្នុងឈាមរបស់អ្នក ក្នុងរយៈពេលមួយឆ្នាំចុងក្រោយនេះ ដែរ​ ឬទេ?

①ទេ/អត់ដឹង　 ②ម្តង　 ③២ដង ④៣ដងឡើង

1. តើអ្នកបានវាស់ជាតិស្ករក្នុងឈាមរបស់អ្នក លើកចុងក្រោយនៅឯណា? ជាមួយអ្នកណា?

①ទេ/អត់ដឹង　②ធ្វើខ្លួនឯងនៅផ្ទះ　③មិត្តអប់រំមិត្តធ្វើអោយនៅផ្ទះខ្លួនឯង ④ប៉ុស្តិ៍ ឬមណ្ឌលសុខភាព

⑤មន្ទីរពេទ្យរដ្ឋ ⑥នៅពេទ្យឯកជន　⑦មណ្ឌលព័ត៌មានជំងឺ　⑧ផ្សេងៗ（　...........................　　）

1. តើអ្នកធ្លាប់បានមូបឈាមធ្វើតេស្តនៅមន្ទីរពិសោធន៍វេជ្ជសាស្រ្ត ក្នុងរយៈពេលមួយឆ្នាំចុងក្រោយនេះ ដែរ​ ឬទេ?

①ទេ/អត់ដឹង　 ②ម្តង　③២ដង ④៣ដងឡើង

1. តើអ្នកបានមូបឈាមធ្វើតេស្តនៅមន្ទីរពិសោធន៍វេជ្ជសាស្រ្ត លើកចុងក្រោយនៅឯណា?

①ទេ/អត់ដឹង　 ②ប៉ុស្តិ៍ ឬមណ្ឌលសុខភាព ③មន្ទីរពេទ្យរដ្ឋ ④នៅពេទ្យឯកជន

⑤នៅពេទ្យឯកជន　 ⑥ផ្សេងៗ（　...........................　　）

**5. ការចំណាយសម្រាប់ការស្វែងរកសេវាព្យាបាល**

1. តើអ្នកធ្លាប់បានចូលដេកពេទ្យ(IPD only)ដែរ​ ឬទេ ក្នុងរយៈពេល ១ឆ្នាំចុងក្រោយនេះ?

①ទេ/អត់ដឹង　 ②ម្តង　 ③២ដង ④៣ដងឡើង

1. តើអ្នកចូលសម្រាកនៅមន្ទីរពេទ្យចុងក្រោយបង្អស់ ប៉ុន្មានថ្ងៃ?

①ទេ/អត់ដឹង　 ②1-3ថ្ងៃ ③4-6ថ្ងៃ　④៧ថ្ងៃឡើង

1. តើអ្នកចំណាយសម្រាប់ធ្វើដំណើរទៅមកមន្ទីរពេទ្យ អស់ប៉ុន្មានសម្រាប់រយៈពេល ១ខែចុងក្រោយនេះ?

①ទេ/អត់ដឹង　 ②ក្រោម ១ដុល្លា　②១ ទៅ ២ ដុល្លា　 ③២ ទៅ៣ ដុល្លា

④៣ ទៅ ៤ដុល្លា　⑤៤ ទៅ ៥ដុល្លា　⑥ចាប់ពី៥ ដុល្លាឡើង

1. តើអ្នកត្រូវចំណាយសម្រាប់សេវាពេលប្រចាំខែ អស់ប៉ុន្មានក្នុងរបស់ពេល១ខែ?

(រួមសេវាពីគ្រោះជំងឺ, សេវាពិនិត្យឈាម, ថ្នាំពេទ្យ)

①ទេ/អត់ដឹង　 ②ក្រោម ១ដុល្លា　 ②១ ទៅ ៣ ដុល្លា　③៣ ទៅ ៥ ដុល្លា

④៥ទៅ ៧ ដុល្លា　⑤៧ ទៅ ១០ ដុល្លា　　 ⑥ចាប់ពី១០ ដុល្លាឡើង

**6. ចំណេះដឹងទាក់ទងនឹងជំងឺទឹកនោមផ្អែម**

1. ខ្ញុំអាចហូបស៊ុតបានច្រើន តាមចំណងឃ្លានរបស់ខ្ញុំ៖

①ត្រឹមត្រូវ　 ②ខុស　 ③មិនដឹង

1. អាចហូបត្រីបានច្រើន តាមចំណងឃ្លានរបស់ខ្ញុំ៖

①ត្រឹមត្រូវ　 ②ខុស ③មិនដឹង

1. អាចហូបសណ្តែកបានច្រើន តាមចំណងឃ្លានរបស់ខ្ញុំ៖

①ត្រឹមត្រូវ ②ខុស　 ③មិនដឹង

1. អាចហូបបាយសបានច្រើន តាមចំណងឃ្លានរបស់ខ្ញុំ៖

①ត្រឹមត្រូវ　 ②ខុស　 ③មិនដឹង

1. អាចហូបនំប៉័ងបានច្រើន តាមចំណងឃ្លានរបស់ខ្ញុំ៖

①ត្រឹមត្រូវ　 ②ខុស　 ③មិនដឹង

1. ធ្វើលំហាត់ប្រាណបានទៀងទាត់ វាជួយគ្រប់គ្រងជំងឺទឹកនោមផ្អែម៖

①ត្រឹមត្រូវ　 ②ខុស　 ③មិនដឹង

1. ការជក់បារី ជក់ស៊ីហ្គា ឬ ថ្នាំចុក គឺធ្វើអោយប៉ះពាល់ដល់ការគ្រប់គ្រងជំងឺទឹកនោមផ្អែម៖

①ត្រឹមត្រូវ　 ②ខុស ③មិនដឹង

1. ពិសារគ្រឿងស្រវឹង គឺធ្វើអោយប៉ះពាល់ដល់ការគ្រប់គ្រងជំងឺទឹកនោមផ្អែម៖

①ត្រឹមត្រូវ　 ②ខុស　 ③មិនដឹង

1. អាក្ការៈស្ពឹក និង ចាក់ឆៀបៗ អាចជារោគសញ្ញានៃបញ្ហាប្រព័ន្ធប្រសាទ៖

①ត្រឹមត្រូវ　 ②ខុស　 ③មិនដឹង

**7. ឥរិយាបថទាក់ទងនឹងជំងឺទឹកនោមផ្អែម**

1. នៅពេលអ្នកមានជំងឺទឹកនោមផ្អែម តើអ្នកគួរស្វែងរកការព្យាបាលវាដែរ ឬទេ?

①បាទ/ចាស៎　 ②ទេ　 ③មិនដឹង

1. តើអ្នកគិតថា ខ្លួនអ្នកអាចមានឥទ្ធិពលលើការគ្រប់គ្រងជំងឺទឹកនោមផ្អែមដែរ​ ឬទេ?

①បាទ/ចាស៎　 ②ទេ　 ③មិនដឹង

**8. ការបដិបត្តិប្រចាំថ្ងៃទាក់ទងនឹងជំងឺទឹកនោមផ្អែម**

1. តើអ្នកអនុវត្តតាម របបអាហារសម្រាប់អ្នកជំងឺទឹកនោមផ្អែមដែរ​ ឬទេ?

①បាទ/ចាស៎　 ②ទេ

1. ​តើអ្នកបាននឹងកំពុងធ្វើលំហាត់ប្រាណជាទៀតទាត់ដែរ ឬទេ សព្វថ្ងៃនេះ

①បាទ/ចាស៎　 ②ទេ

1. តើអ្នកកំពុងតែប្រើប្រាស់ថ្នាំជក់ដែរ​ ឬទេ?

①បាទ/ចាស៎　 ②ទេ

1. តើពីមុនមក អ្នកជក់បារីដែរ ឬទេ? (ទោះបីពេលនេះ មិនជក់ក៏ដោយ)

①បាទ/ចាស៎　 ②ទេ

1. តើជាទំលាប់ប្រចាំថ្ងៃ អ្នកពិសារគ្រឿងស្រវឹងដែរ​ ឬទេ?

①បាទ/ចាស៎　 ②ទេ

1. តើអ្នកបានប្រយ័ត្ន នៅរាល់ពេលកាត់ក្រចកជើងដែរ ឬទេ?

①បាទ/ចាស៎　 ②ទេ

1. តើអ្នកធ្លាប់បានប្រឹក្សាយោបល់ជាមួយ អ្នកធ្វើការខាងសុខភាព អំពីជំងឺទឹកនោមផ្អែមរបស់អ្នក ក្នុងរយៈពេល ៣ខែចុងក្រោយដែរ ឬទេ? (រាប់ទាំង មិត្តអប់រំមិត្ត, អ្នកស្ម័គ្រចិត្តសុខភាពសហគមន៍, គ្រូពេទ្យជំនាញ, គ្រូពេទ្យ ឬអ្នកជំនាញខាងរបបអាហារ)

①បាទ/ចាស៎　 ②ទេ

**9. កាលានុវត្តភាពក្នុងការទទួលបានព័ត៌មានអំពីសុខភាព**

1. តើអ្នកធ្លាប់បានចូលរួមក្នុងការបង្រៀនជាក្រុមជាមួយមិត្តអប់រំមិត្ត(តាមដាននៅផ្ទះមិត្តអប់រំមិត្ត)​ ក្នុងរយៈពេលមួយឆ្នាំចុងក្រោយដែរ ឬទេ?

①អត់/មិនដឹង ②ម្តង ③២ដង ④៣ដង 　⑤៤ដង ⑥៥ដង　 ⑦៦ដង

1. តើអ្នកទទួលបានព័ត៌មានអំពីសុខភាព ក្នុងរយៈពេលមួយឆ្នាំចុងក្រោយនេះ ជាចម្បងតាមរយៈណា?

①គ្រួសារ　②មិត្តភក្តិ　③មិត្តអប់រំមិត្ត　④អ្នកស្ម័គ្រចិត្តតាមសហគមន៍　⑤មណ្ឌលសុខភាព

⑥មន្ទីរពេទ្យរដ្ឋ　⑦ពេទ្យឯកជន ⑧វិទ្យុ　 ⑨ទូរទស្សន៍ ⑩ផ្ទាំងរូបភាព/កាសែត/ខ័ត្តបណ្ណនានា

⑪ផ្សេងៗ ..........

1. តើប្រភពណាមួយដែលទុកចិត្តបំផុត ក្នុងការទទួលបានព័ត៌មានអំពីសុខភាព?

①គ្រួសារ　②មិត្តភក្តិ　③មិត្តអប់រំមិត្ត　④អ្នកស្ម័គ្រចិត្តតាមសហគមន៍　⑤មណ្ឌលសុខភាព

⑥មន្ទីរពេទ្យរដ្ឋ　⑦ពេទ្យឯកជន ⑧វិទ្យុ　 ⑨ទូរទស្សន៍ ⑩ផ្ទាំងរូបភាព/កាសែត/ខ័ត្តបណ្ណនានា

⑪ផ្សេងៗ ..........

***ការសំភាសន៍ ត្រូវបានបញ្ចប់ត្រឹមនេះ សូមអរគុណច្រើន ចំពោះការចំណាយពេលរបស់លោកអ្នក!***

**10. ផ្នែកត្រូវសម្លងទិន្នន័យពីប្រព័ន្ធទិន្នន័យ**

1. កាលបរិច្ឆេទ ចុះឈ្មោះជាសមាជិកបណ្តាញមិត្តអប់រំមិត្ត

ខែ............... ឆ្នាំ..................

1. លទ្ធផលតេស្តស្រ្តីបទឹកនោម នៅពេលអ្នកជំងឺចុះឈ្មោះជាមួយបណ្តាញមិត្តអប់រំមិត្ត

①－　②±　③＋　④＋＋　⑤＋＋＋　⑥＋＋＋＋

1. កាលបរិច្ឆេទ តេស្តស្រ្តីបទឹកនោម​ ចុងក្រោយបំផុត

ខែ............... ឆ្នាំ..................

1. លទ្ធផលតេស្តស្រ្តីបទឹកនោម ចុងក្រោយបំផុត

①－　②±　③＋　④＋＋　⑤＋＋＋　⑥＋＋＋＋

1. កាលបរិច្ឆេទ ថ្លឹងទំងន់ ចុងក្រោយបំផុត

ខែ............... ឆ្នាំ..................

1. លទ្ធផលវាស់ កម្ពស់ និង ទំងន់ ចុងក្រោយបំផុត

កំពស់៖ ..................សង់ទីម៉ែត្រ, ទំងន់.............គីឡូក្រាម

1. កាលបរិច្ឆេទ តេស្តឈាមក្នុងមន្ទីរពិសោធន៍ ចុងក្រោយបំផុត

ខែ............... ឆ្នាំ..................

1. លទ្ធផលតេស្តឈាមក្នុងមន្ទីរពិសោធន៍ ចុងក្រោយបំផុត
